# A Method for Settlement Detection of the Transmission Line Tower under Wind Force

**DOI:** 10.3390/s18124355

**Published:** 2018-12-10

**Authors:** Xinbo Huang, Yu Zhao, Long Zhao, Luya Yang

**Affiliations:** 1 School of Electronics and Information, Xi’an Polytechnic University, Xi’an 710048, China; zhaoyu@xpu.edu.cn (Y.Z.); yangluya@xpu.edu.cn (L.Y.); 2 School of Electro-Mechanical Engineering, Xidian University, Xi’an 710070, China; zhaolong@xpu.edu.cn

**Keywords:** transmission tower, settlement, wind force, acceleration, modal frequencies

## Abstract

In view of the settlement problem of transmission tower foundation, the vibration characteristics of transmission towers under wind force are measured experimentally. In this paper, the 110 kV cat head transmission tower of Xi’an Polytechnic University is measured and analyzed. Firstly, the acceleration sensor and meteorological sensor are installed on the tower to collect the vibration response and environment parameters of the tower in real time. Then, an experiment platform is built to simulate the tower settlement, and the vibration response of the tower after settlement is measured in time. Finally, the low-order modal frequencies of the transmission tower before and after settlement under wind force load are extracted by stochastic subspace identification (SSI), and the relationship between modal frequencies of different modes is analyzed via temperature correction. By comparison and analysis, it is obvious that the X-direction modal frequencies before and after settlement under natural wind load are changed, and the change rate increases with the increase of settlement displacement, which can be used as effective evidence for judging the settlement of transmission tower foundation.

## 1. Introduction

In electrical power systems, the transmission tower is an integral component of power transmission, and its safe operation is an important factor. Terrain conditions of the transmission tower are complex, where often exist phenomena such as displacement, inclination, cracking and subsidence [1]. In particular, the settlement tilt of a tower can be caused by severe natural environmental conditions (such as rain, snow, wind and so on) or man-made destruction and other factors, which may even cause the tower collapse. Because the stress of tower foundations will change when settlement occurs [2], many scholars have tried to solve this problem with experimental simulation, and a significant amount of theoretical research has been undertaken. Some researchers have taken the tower line system as the research object and analyzed the vibration characteristics of the tower line system under the action of wind [3,4]. The finite element analysis method has often been used to analyze the dynamic characteristics and stability in wind conditions in the case of the complex structure of the tower [5,6]. Some scholars have conducted nonlinear buckling analysis of the tower line system, determined the critical wind load and analyzed the dynamic characteristics of the tower line system under different wind loads. It has been found that low modal frequency is a useful indicator for predicting the occurrence of structural instability [7]. In addition, some scholars have carried out wind tunnel tests on the EHV (Extra-high Voltage) transmission tower line system and studied the dynamic characteristics of the system under different wind speeds [8]. However, the method of mechanical analysis is usually used to study the response of the tower to wind, and there are few methods to identify the abnormal structure of the tower.

Structural health monitoring technologies are widely used in structural damage detection, including for electric power tower structures. The method of measuring tower inclination with an inclination sensor has been widely used [9]. However, this method can only indirectly reflect the stress of the large deformation of a tower, and parameters of load balance, stealth faults (such as the small deformation of the tower) or the yield failure of the local rod cannot be found in time. Some researchers realized the remote monitoring of tower structure damage by installing strain sensors on the transmission tower [10]. Although the resistance strain gauge can identify the force change of the rod, there are still some problems, such as complex attachment processes, susceptibility to rust and electromagnetic interference on the measuring circuit. Therefore, some researchers use grating fiber strain sensors to improve the range and accuracy of the measurement [11,12]; however, the development of this method is limited by installation quantity, installation location and the direction of the sensor.

The technology of modal identification has been studied in the field of structural health monitoring. In references [13,14], passing vehicles on a reinforced concrete bridge vibrated the bridge and the damage to the bridge was studied by modal identification. In reference [15], a wind turbine blade is vibrated by an excitation device and the damage to the wind turbine blade is identified by the modal technique, and the position of the damage or length of the crack is determined. Under the action of wind load, the transmission conductor will generate vertical vibration due to the presence of the Karman vortex. In reference [16], the modal analysis of the transmission line is carried out to realize the detection of the broken conductor of the transmission line. However, there is almost no modal analysis study for transmission towers. Transmission tower structures are completely different from reinforced concrete bridges, wind turbines and transmission lines. Their vibrations are multi-directional, so existing technologies cannot be applied directly to iron towers. However, iron towers prone to accidents are often in unattended areas such as in mountains and hills. The environmental impact in these areas are small, which can greatly reduce the workload of research. Therefore, a method for settlement detection of the transmission line tower under wind force is proposed in this paper. By installing acceleration sensors and meteorological sensors on the transmission tower, the system can collect vibration acceleration signals of the tower and related parameters (wind speed, wind direction, temperature) of the surrounding environment in real time. The modal frequency of the tower can be identified through modal analysis under wind force, by which it can be judged whether tower settlement occurs. Finally, the system is tested on the 110 kV cat head transmission tower of Xi’an Polytechnic University. The measured vibration data are analyzed by stochastic subspace identification (SSI) and modal parameters are modified via the ambient temperature. The result shows that the system can accurately obtain the structural information of the tower by identifying its modal parameters, which can be used as effective evidence for judging the settlement of the transmission tower foundation.

## 2. Principle and Experiments

### 2.1. Principle

There are many types of transmission towers with different complex structures and stiffness. Moreover, even the same tower has different degrees of stiffness due to the installation process. However, if we can find a single parameter to reflect the structure of the tower system and use it as the reference to the normal structure, the structure can be considered to have a fault when the parameter changes significantly. The transmission tower vibration has multiple degrees of freedom under wind load. According to the dynamic equation of a linear system with N degrees of freedom, the vibration equation of the tower can be expressed as follow:

(1) $$M\overset{••}{x}(t)+C\overset{•}{x}(t)+Kx(t)=f(t)$$

where M, C, K are the mass, damping and stiffness matrices of the tower structure; x(t) is the displacement response time history vector; and f(t) is the excitation time history vector, which is also the wind load action. In this operation, other factors can also influence the vibration of power transmission towers. From the motion equation, although the excitation on the right side of the equation changes, the stiffness and mass of the tower do not change. Different excitations have different frequencies, which can excite different modal. However, these have little effect on the modal parameters of the system. The experimental environment of this paper is the 110 kV three-tower and two-gear transmission lines of Xi’an Polytechnic University. It mainly considers the vibration response of the transmission tower under wind excitation. When transmission tower settlement occurs, especially uneven settlement, the stiffness matrix K and damping matrix C will change and the modal frequency of the corresponding tower system will also change. According to this characteristic, we can monitor whether tower settlement occurs by the modal frequency of the tower system.

### 2.2. Experiments

#### 2.2.1. Experimental Tower

**Figure 1:**
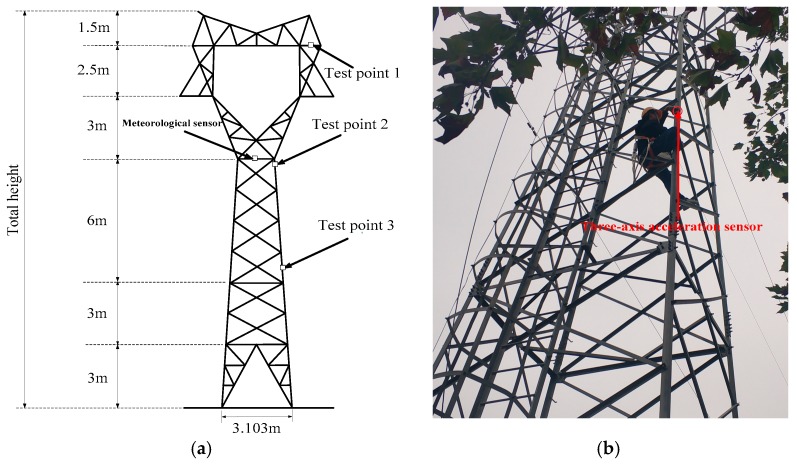
ZM-110 kV cat head tower: (**a**) transmission tower model; (**b**) installation of the monitoring device.

The transmission tower studied in this paper is a ZM-110 kV cat head tower which is built in Xi’an Polytechnic University. The LGJ-95/15 conductors are installed on the tower and the direction of the lines are north-south. The cat head tower’s total height is 19 m and the root is 3.103 m, as shown in Figure 1a.

#### 2.2.2. Sensor Installation

One meteorological sensor and three three-axis acceleration sensors were installed on the transmission tower so as to measure the meteorological parameters around the tower and the vibration signals at three measuring points. The layout of the points is shown in Figure 1a. The tower will vibrate when the natural wind exerts load excitation on the tower. Because the stiffness of the tower is high, the natural wind load is not very strong in general. Moreover, the degrees of freedom of the tower legs are constrained and vibration near the tower legs is not obvious. On the contrary, vibration near the tower top is easier to identify. Thus, the measuring points are selected to be the junction of the tower cross arm and the ground support, the tower mouth, and the tower body main material at the height of 7.5 m from the ground, respectively [17]. Selecting these positions as the measuring points is helpful to precisely extract the vibration acceleration of the tower. The three-axis acceleration sensor has a magnetic force on one side, allowing it to be firmly attached to the angled steel of the tower, as shown in Figure 1b.

#### 2.2.3. Real-Time Measurement

**Figure 2:**
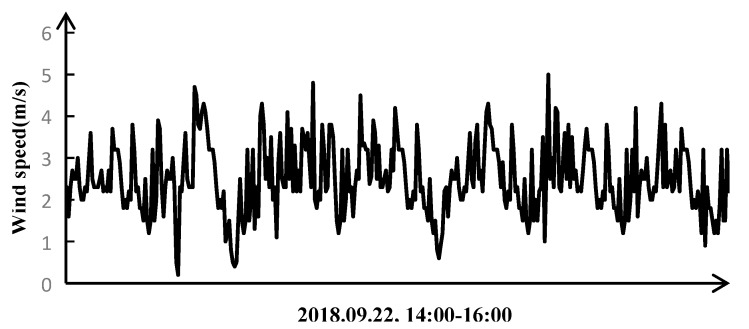
Wind speed time history curve during the test period.

In order to prove that the modal frequencies of the transmission tower system can be well extracted by modal identification, the transmission tower in this paper was measured and analyzed at 14:00 on 22 September, 2018. At the same time, the tower settlement experiment platform was built to simulate the settlement process of single tower foundation, and the low-order modal frequencies of the transmission tower system before and after settlement were extracted and analyzed. The weather was sunny, with a south-east wind and wind level of 3–4. The sampling frequency of the three-axis acceleration sensor was set to 200 Hz and the sampling time was 30 min because the low-order modal frequency of the tower is within 1–15 Hz [18]. Figure 2 shows the wind speed history curve measured by the meteorological sensor during the test period.

**Figure 3:**
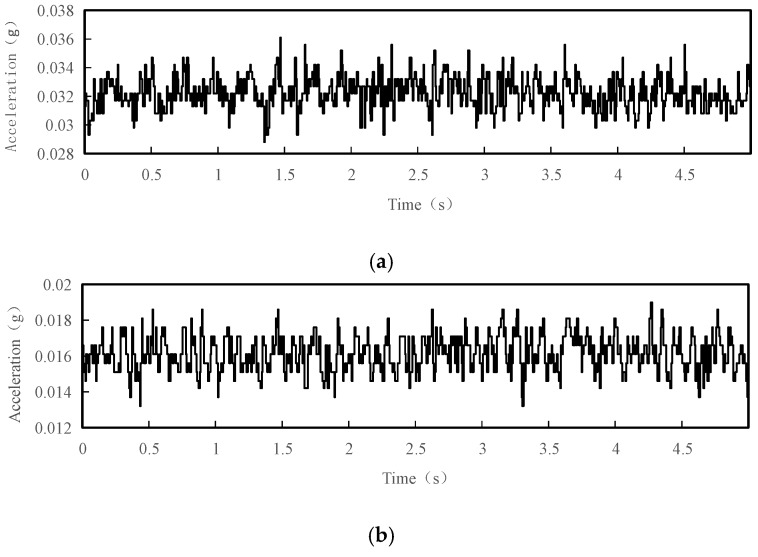

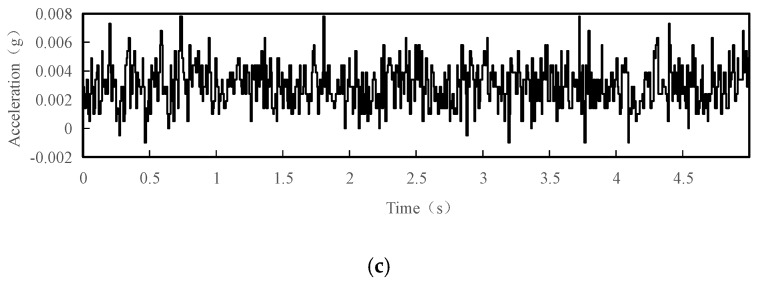
Acceleration time history curves with 5 s interval of the test point 1 under normal conditions: (**a**) acceleration time history curve in the X direction; (**b**) acceleration time history curve in the Y direction; (**c**) acceleration time history curve in the Z direction.

Considering that the tower vibration is not a free vibration in single direction, the three-axis acceleration sensor was used to monitor the acceleration signals in the X, Y and Z directions of the tower in this paper; these signals were processed and analyzed separately. Figure 3 shows the acceleration time history curves for a 5 s interval in the X, Y and Z directions of test point 1 under normal conditions.

**Figure 4:**
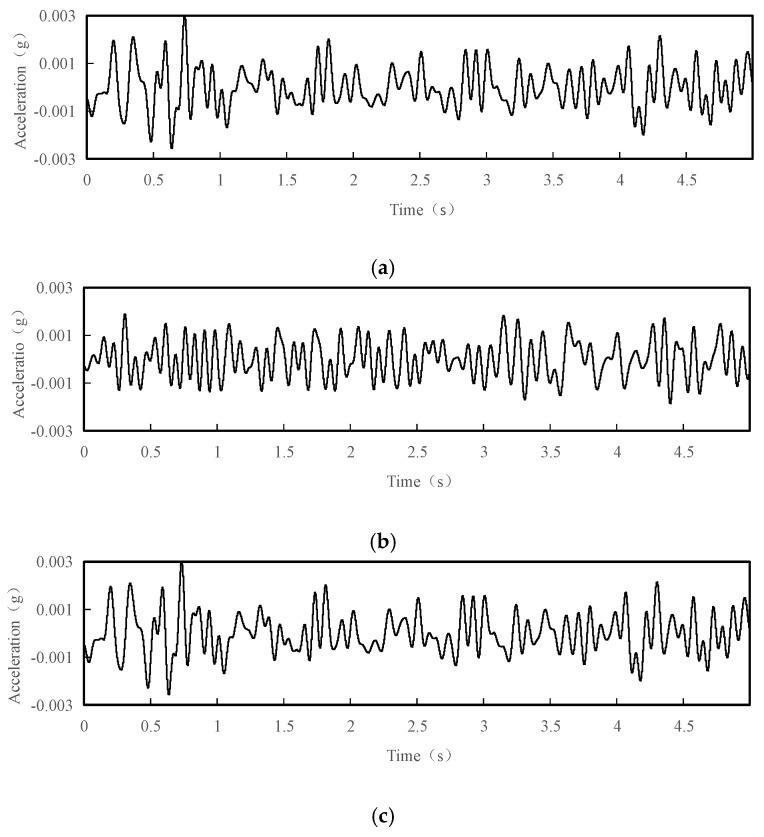
Acceleration time history curves with interval of 5 s of the test point 1 after filtering under normal conditions: (**a**) acceleration time history curve in the X direction after filtering; (**b**) acceleration time history curve in the Y direction after filtering; (**c**) acceleration time history curve in the Z direction after filtering.

The collected vibration signals needed to be pre-processed before subsequent analysis. A low-pass filter with cut-off frequency of 15 Hz is used to identify the low-order vibration frequency characteristics of the tower, and high-order vibration frequencies with small amplitudes are neglected. Figure 4 shows the acceleration time history curves for a 5 s interval after filtering of the test point 1 under normal conditions.

### 2.3. Vibration Analysis

Stochastic subspace identification (SSI) is a new method for linear system identification developed in recent years. It can extract model parameters directly from the corresponding output signals of the environmental excitation rather than from artificial excitation. The key of this method is to determine the order of the system. A stabilization diagram is a novel approach which can be used for modal identification in the case of strong noise [19]. In this paper, stochastic subspace identification is used to analyze the acceleration signal after filtering, and the stabilization diagram is used for modal identification of the system.

**Figure 5:**
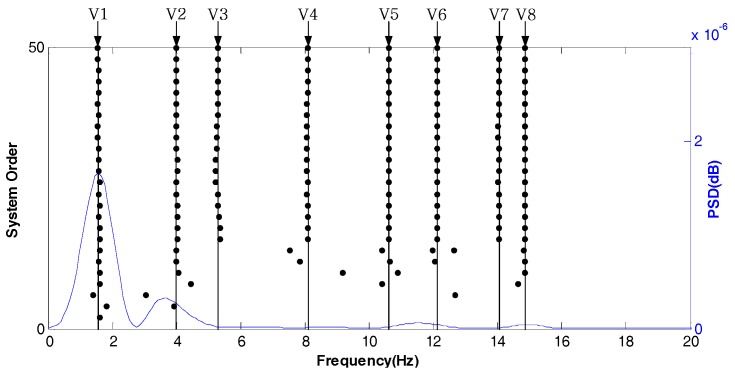
Stabilization diagram in the X direction of test point 1 under normal conditions.

Figure 5 shows the stabilization diagram in the X direction of test point 1 under normal conditions. Its abscissa represents the modal frequency and V1–V8 represent the eight modes of the tested tower system, respectively. According to the requirements of the stability axis, $\left|\frac{f_{i}-f_{i+1}}{f_{i}}\right|<[\Delta f_{e}]$, where i is the modal order, f is the modal frequency and [•] is the modal frequency limit parameter. The stability of the system can be determined by the intensity of the points on the stable axis. As shown in Figure 5, even if the power spectral density (PSD) is not prominent, the eight modes in the X direction can be well analyzed and the modal frequency is also stable.

Table 1 lists the modal frequencies in the X direction of test points under normal conditions calculated by SSI. Because of the influence of the installation position and the wind condition of the acceleration sensor, the modal frequencies in some orders of the three test points change slightly. However, the overall frequency identification results are relatively good. In the following analysis, we use the average value of the three test points for analysis.

**Figure 6:**
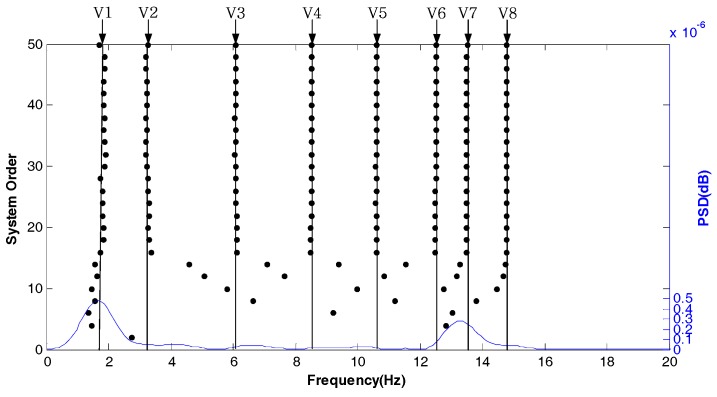
Stabilization diagram in the Y direction of test point 1 under normal conditions.

Figure 6 shows the stabilization diagram in the Y direction of test point 1 under normal conditions. It can be seen that the modal frequencies of each order are relatively stable.

Table 2 shows the modal frequencies in the Y direction of the test points under normal conditions calculated by SSI. The modal frequencies of three test points are less consistent than those in the X direction, but the modal frequencies are stable.

**Figure 7:**
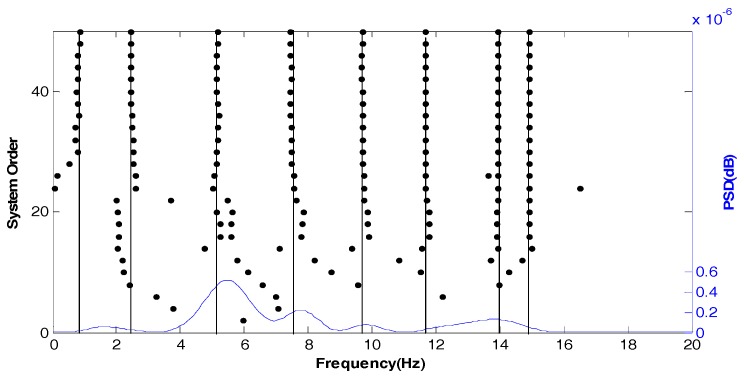
Stabilization diagram in the Z direction of the test point 1 under normal conditions.

Figure 7 shows the stabilization diagram in the Z direction of test point 1 under normal conditions. The modal frequency of the Z direction is very unstable. If the modal parameters of the Z direction are used as evidence for tower settlement, error would be introduced.

## 3. Field Test and Discussion

### 3.1. Experiment and Analysis of Tower Foundation Settlement

Transmission tower settlement is caused by its foundation and underground soil, which is uniform or uneven. Under uneven settlement, the height difference of the four tower foundations may cause a large change in the force of the transmission tower, which also causes the stiffness and the modal frequency of the tower system to change. Since a damage test cannot be carried out, the process of tower foundation settlement cannot be readily simulated. Therefore, a hydraulic jack was used to raise the tower foundation, which alters the height of the tower foundation and verifies the feasibility of the proposed method.

#### 3.1.1. Tower Foundation Settlement

**Figure 8:**
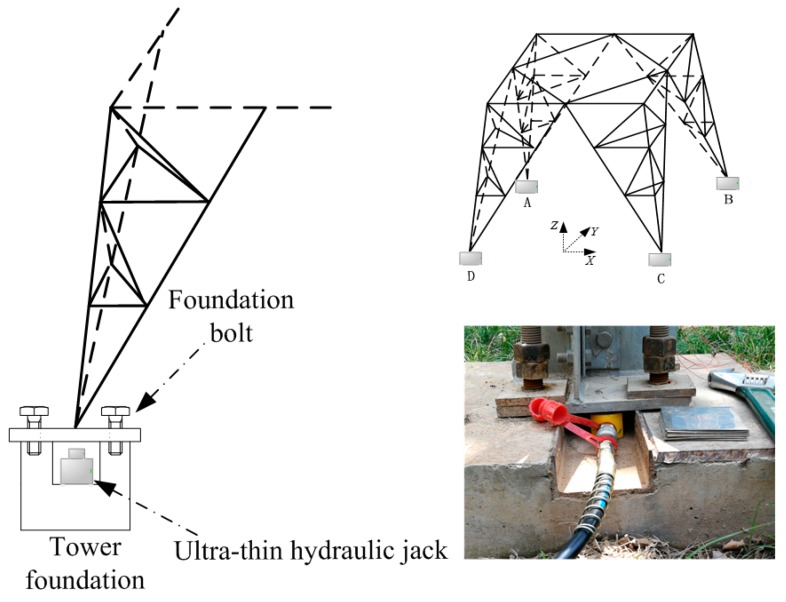
Tower foundation settlement experiment platform.

The anchor bolts of the ZM-110 kV cat tower in Xi’an Polytechnic University are outlying, which is convenient for building the settlement experimental platform. Figure 8 is a schematic diagram of the experimental platform. Before tests, the tower foundation must be cleaned to ensure the cleanliness of the tower foundation as much as possible, so as not to introduce unnecessary errors. Firstly, the ultra-thin hydraulic jack can be placed at the bottom of the tower foundation by releasing the anchor bolts. Then, the tower foundation can be lifted by raising the ultra-thin hydraulic jack, and recording the height of the tower foundation from the groove bottom as $h_{x}$. When the tower is installed and designed, the initial height of the tower foundation and the groove bottom is recorded as $h_{0}$. Finally, the longitudinal displacement $\Delta h=h_{x}-h_{0}$ can be obtained. Because the displacement of the ultra-thin hydraulic jack is limited, 2-mm stainless-steel plates can be added to the groove bottom without withdrawing the last measuring jack. The longitudinal displacement can be increased by applying longitudinal displacement with another hydraulic jack.

#### 3.1.2. Settlement Experiment Analysis

In order to determine which direction of modal frequency under natural load can provide better evidence for settlement monitoring, we use the settlement experimental platform to settle 4 mm and 6 mm of tower foot A, and the recorded settlement data are processed and analyzed.

In Table 3, the average modal frequencies and the change rate in the X direction of tower foot A before and after settlement are recorded. $\omega_{0}$, $\omega_{4}$ and $\omega_{6}$ represents the modal frequencies in settlements of 0 mm, 4 mm and 6 mm, respectively. $\frac{\left|\omega_{4}-\omega_{0}\right|}{\omega_{0}}\%$ and $\frac{\left|\omega_{6}-\omega_{0}\right|}{\omega_{0}}\%$ represent the change rate of the modal frequencies of the tower system in settlements of 4 mm and 6 mm, respectively. It can be seen that the modal frequency of the tower experienced an obvious change after 4 mm settlement; the change rates of the modal frequency of six modes exceeded 3% and the change rate of the third mode exceeded 10%. When settling 6 mm, the change rate is further increased, with the change rates of five modes exceeding 5%. The repeated tests show that the results are stable. It can be seen that the X direction low-order modal frequencies of the tower system can be used as evidence for tower foundation settlement.

**Figure 9:**
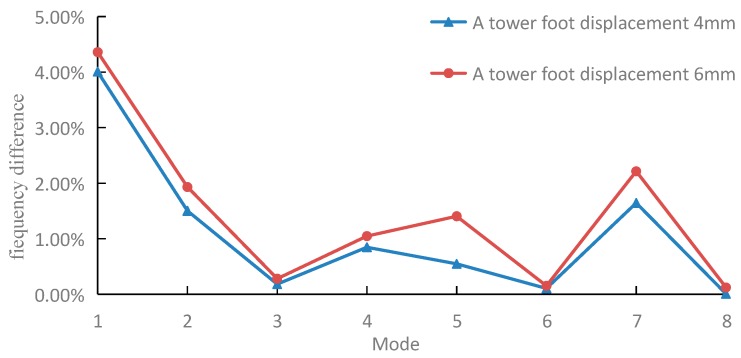
Change rate of modal frequency in the Y direction of the tower foot A before and after settlement.

Figure 9 is a graph of the modal frequency change rate and order in the Y direction of tower foot A before and after settlement. After settlement, the modal frequency of the first mode changes clearly, while changes for other modes are less obvious. Thus, the modal frequency in the Y direction is not suitable as evidence of tower foundation settlement under natural load.

**Figure 10:**
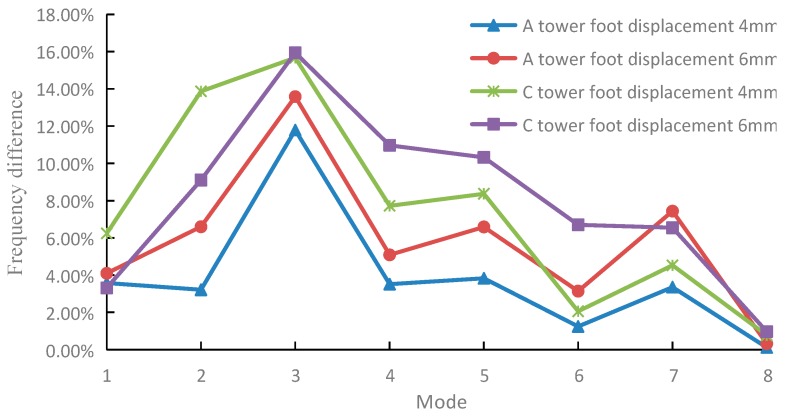
Change of modal frequency in the X direction of the A and C tower feet before and after settlement.

In order to ensure that the low-order modal frequency in the X direction can be used as evidence for tower foundation settlement, we carried out the settlement experiments of 4 mm and 6 mm on tower foot C. Figure 10 is the change rate of the modal frequency in the X direction before and after the settlement of tower foot A and C. The variation of the modal frequencies of each mode of tower foot C after settlement is consistent with that of tower foot A after settlement, which verifies the conclusion that the low-order modal frequency in the X direction can be used to indicate tower foundation settlement. In addition, it can be seen from Figure 10 that 3rd, 5th, and 7th mode frequencies in the X direction of the tower system change clearly, and the change rate of the modal parameters are effective to judge whether settlement occurs during the actual monitoring process.

### 3.2. Influence of Temperature on Modal Frequency

**Figure 11:**
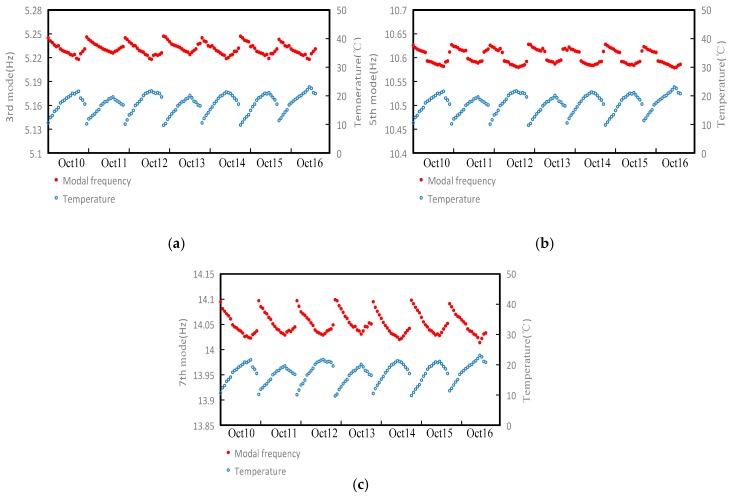
Curves of ambient temperature and modal frequency: (**a**) curve of ambient temperature and 3rd modal frequency; (**b**) curve of ambient temperature and 5th modal frequency; (**c**) curve of ambient temperature and 7th modal frequency.

A large number of studies have shown that the ambient temperature has a great impact on the dynamic characteristics of the structure, which is the most important factor. The modal frequency fluctuations caused by changes of normal temperature can obscure the frequency changes caused by minor structural damage, and even cause misunderstanding of the structural dynamic properties [20]. Considering the effect of the temperature on the modal frequency of the tower system, a one-week test was carried out from 10 October 2018. The ambient temperature and modal frequencies of the 3rd, 5th, and 7th in the X direction of the tower were recorded every 30 min from 9:00 am to 18:00 pm. Figure 11 shows the relationship between the modal frequency and the temperature change over the seven days. It can be seen that the modal frequency is negatively correlated with the ambient temperature.

**Figure 12:**
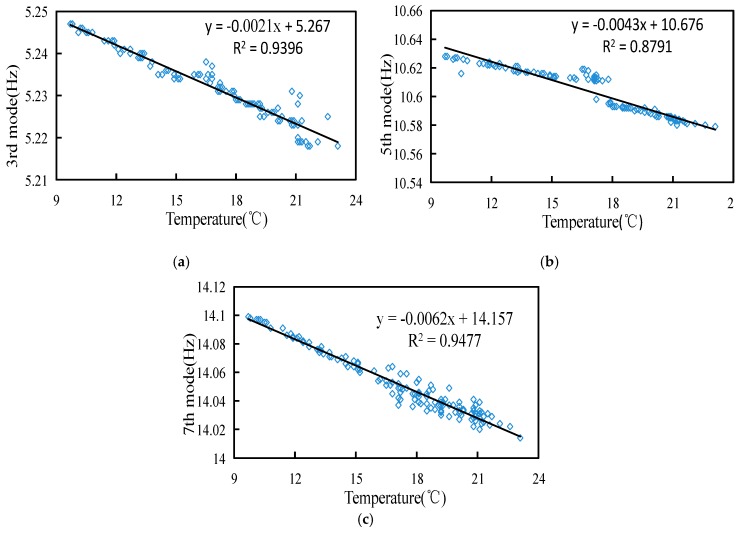
Fitted curves of ambient temperature and modal frequency: (**a**) fitted curve of ambient temperature and 3rd modal frequency; (**b**) fitted curve of ambient temperature and 5th modal frequency; (**c**) fitted curve of ambient temperature and 7th modal frequency.

In order to reduce the influence of ambient temperature on measurements, we fitted the ambient temperature and the modal frequencies of the 3rd, 5th and 7th mode of the tower. The obtained correction coefficient is used to reduce the error caused by the ambient temperature. Figure 12 shows the fitted curves of the ambient temperature and the modal frequency. The variation of the modal frequency is linear with respect to the variation of the ambient temperature.

## 4. Monitoring Technology Realization

### 4.1. System Structure

**Figure 13:**
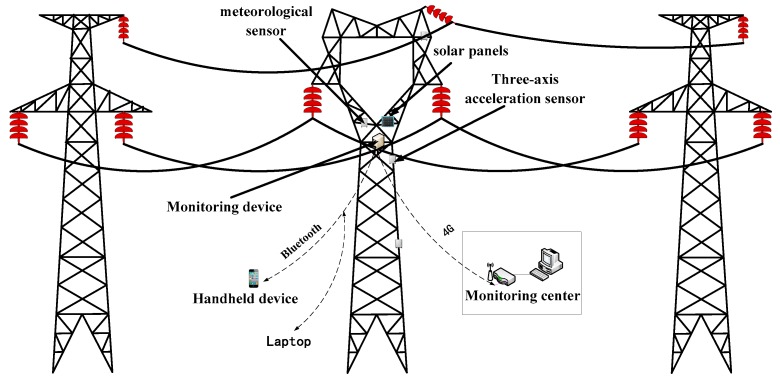
System structure.

In order to realize the on-line monitoring of the tower settlement, a monitoring system for transmission tower settlement is designed. The system shown in Figure 13 mainly includes five parts: three-axis acceleration sensors, monitoring device, meteorological sensors, power supply module and monitoring center. The outer shell of the monitoring device is a stainless-steel rigid box, which is installed on the cross arm of the tower to obtain the vibration signal of the tower, the wind speed and direction, and the temperature around the tower in real time. The monitoring center communicates with the monitoring device by 4G wireless communication, which can receive the vibration data of the tower in real time or regular time. The filtering algorithm, stochastic subspace identification (SSI) modal identification model and tower settlement fault diagnosis model are embedded via the expert software of the monitoring center. Using the filtering algorithm to process the measured data is helpful to improve the vibration frequency identification accuracy of the transmission tower. The SSI identification model can extract the low-order frequencies of the transmission tower system from the received vibration data. The tower settlement fault diagnosis model determines whether a tower settlement accident has occurred by comparing the modal parameters with the tower without settlement and gives an alarm in case of an accident. At the same time, the operator can obtain the accident information at initiation and take timely corrective measures to prevent further development of the accident. In field maintenance or testing, the operator can obtain the monitoring device data through Bluetooth by using a handheld device or laptop. Solar power and battery are used to provide electricity for the whole system. The best installation angle of solar panels is determined by actual operating conditions, and the battery is located in the stainless-steel rigid box.

### 4.2. Monitoring Device

**Figure 14:**
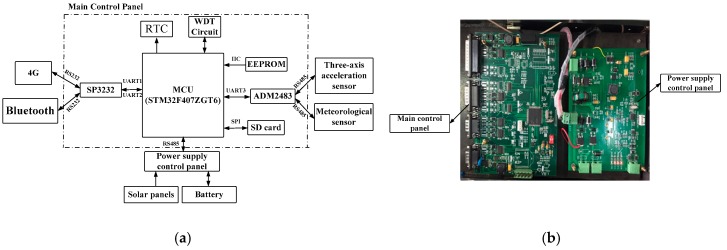
Monitoring device: (**a**) principle diagram of the monitoring device, RTC(Real-Time Clock),WDT(Watchdog Timer); (**b**) main control and power supply control panels.

The principle diagram of the monitoring device is shown in Figure 14a, including the main control unit (MCU), power module, communication unit (4G/Bluetooth/RS485) and sensors. The power module is composed of solar panels, batteries and controllers, which provide power for the whole system. It has low power consumption and can last for more than 30 days in rainy weather. Sensors include the three-axis accelerometer sensor and the meteorological sensor. The three-axis acceleration sensor is used to collect the vibration response of the tower and the meteorological sensor is used to measure the environmental parameters around the tower. The monitoring device sends the measurement data to the monitoring center and receives the commands from the monitoring center, which include time setting, sampling interval, sampling duration, monitoring device initialization, etc. Figure 14b shows the main control and power supply control panels.

### 4.3. Implementation Process

In reference [21], the minimum critical displacement of the 110 kV cat head tower is 7.5 mm when the settlement is simulated by finite element analysis, and stress distribution is verified by the method of affixing the grating fiber strain gauge and artificially simulating transmission tower sink. Based on above reasons, the threshold value should be less than the critical displacement value of a single tower and considered as a certain margin. In this paper, when the settlement was 4 mm and 6 mm, the maximum change rate of the modal frequency was 11.787% and 13.596% respectively. Considering the error of the artificial simulation settlement and the actual settlement, for the 110 KV cat head tower studied in this paper, the maximum modal change rate is 10%, which is used as the threshold of the monitoring device.

**Figure 15:**
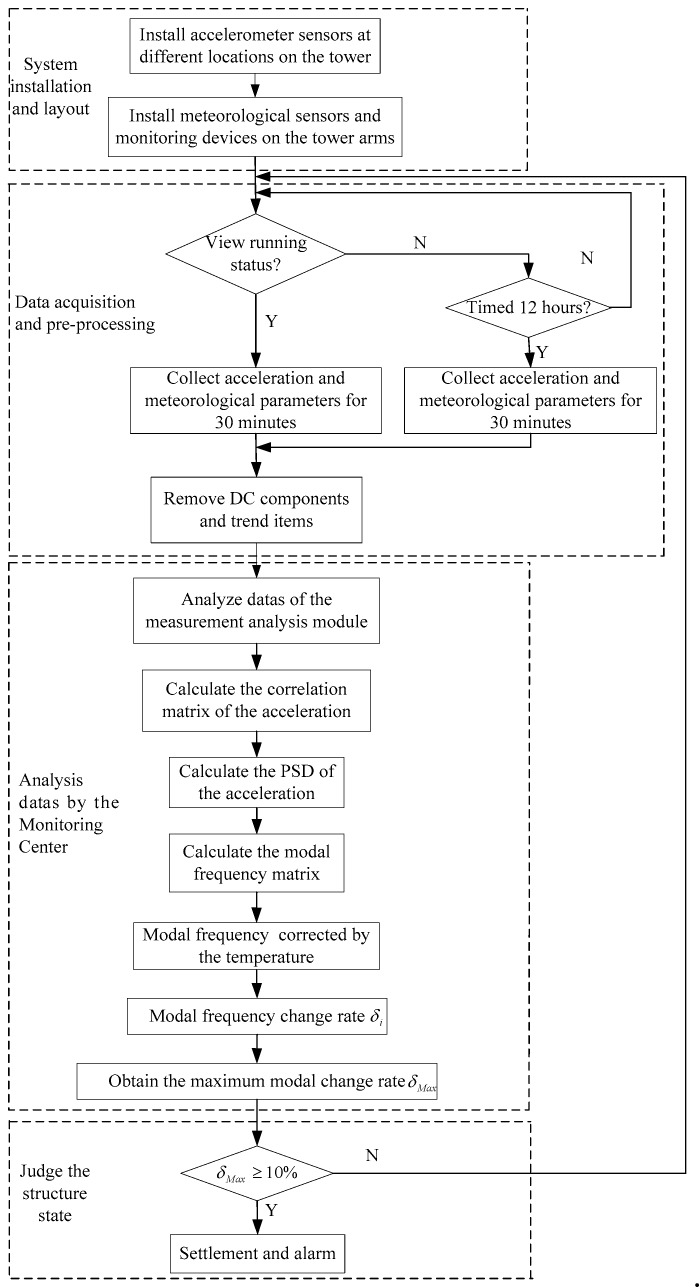
Flow chart of the system.

Figure 15 shows a flow chart of the system. After the installation of the device, ensuring that the tower settlement does not occur within one day, the low-order modal frequency matrix $\omega_{0}$ of the tower system measured on the first day is used as a parameter when the tower structure is intact. The device can collect the environmental parameters around the acceleration signal of the tower system in real time or at a fixed time. The acquired signal is filtered to remove DC (direct current) components and trend items. The monitoring center analyzes the received signal and calculates the correlation function matrix of the acceleration and the PSD. The stochastic subspace is combined with the stable graph method to obtain the modal frequency matrix $\omega_{i}$ of the system. Considering the effect of the temperature on the system, the obtained modal frequency is corrected by the temperature, which is ${\hat{\omega }}_{i}$. The modal frequency change matrix is calculated as $\delta_{i}=\frac{\left|{\hat{\omega }}_{i}-\omega_{0}\right|}{\omega_{0}}\%$, and the largest value $\delta_{Max}$ in the modal frequency matrix $\delta_{i}$ is obtained, $\delta_{Max}=Max\{\delta_{i}\}$, to determine if the $\delta_{Max}$ is exceeded. If it is exceeded, then an alarm is issued.

## 5. Conclusions

In this paper, a method for settlement detection of the transmission line tower under wind force is proposed. Through the measurement and experimental analysis of the ZM-110 kV cat tower of Xi’an Polytechnic University, the following conclusions are drawn.

The low-order modal frequencies in the X and Y directions of the transmission towers under natural load can be extracted by stochastic subspace identification (SSI) and stabilization diagram.

The artificial simulation of the tower settlement shows that the low-order modal frequencies of the different tower feet in the X direction before and after settlement are obvious, and the change rate increases with the increase of the settlement displacement. Therefore, under the action of the natural load, the change of the low-order modal frequency in the X direction can be used as a basis to detect whether the transmission tower is settled.

The experiment found that the ambient temperature has a certain influence on the modal frequency of the tower system, and the modal frequency has a negative correlation with the temperature change. The temperature and modal frequency curves were fitted, and it was found that the change of the modal frequency is linear with the change of the ambient temperature. The coefficients of the modal frequency and temperature change were determined.

This paper studies the dynamic response of transmission towers under wind excitation and considers the effects of the ambient temperature. However, there are other sources of vibration in some areas, such as falling rocks, which interact with the wind to complicate the vibration response of the tower. Next, we will study modal parameter identification under the influence of multiple excitation.

## Figures and Tables

**Table 1:** Modal frequency in the X direction of the test points under normal conditions.

	1st Mode	2nd Mode	3rd Mode	4th Mode	5th Mode	6th Mode	7th Mode	8th Mode
Test point 1	1.538	3.987	5.227	8.006	10.590	12.100	14.030	14.830
Test point 2	1.538	3.987	5.224	8.000	10.611	12.101	14.030	14.830
Test point 3	1.538	3.987	5.227	8.003	10.612	12.008	14.029	14.761
Average	1.538	3.987	5.226	8.003	10.611	12.100	14.030	14.807

**Table 2:** Modal frequency in the Y direction of the test points under normal conditions.

	1st Mode	2nd Mode	3rd Mode	4th Mode	5th Mode	6th Mode	7th Mode	8th Mode
Test point 1	1.698	3.260	6.076	8.503	10.601	12.510	13.503	14.761
Test point2	1.694	3.262	6.075	8.497	10.611	12.501	13.500	14.760
Test point 3	1.698	3.264	6.078	8.504	10.612	12.511	13.499	14.761
Average	1.697	3.262	6.076	8.501	10.608	12.507	13.501	14.761

**Table 3:** Average modal frequency and change rate in the X direction of tower foot A before and after settlement.

	$\mathrm{Settling}\;0\;\mathrm{mm}\;(\omega_{0})$	$\mathrm{Settling}\;4\;\mathrm{mm}\;(\omega_{4})$	$\frac{\left|\omega_{4}-\omega_{0}\right|}{\omega_{0}}\%$	$\mathrm{Settling}\;6\;\mathrm{mm}\;(\omega_{6})$	$\frac{\left|\omega_{6}-\omega_{0}\right|}{\omega_{0}}\%$
1st mode	1.538	1.593	3.576	1.601	4.096
2nd mode	3.987	3.859	3.217	3.724	6.596
3rd mode	5.226	5.842	11.787	5.936	13.596
4th mode	8.003	7.722	3.511	7.596	5.086
5th mode	10.611	10.205	3.826	9.912	6.588
6th mode	12.100	11.950	1.240	11.720	3.140
7th mode	14.030	13.560	3.350	12.987	7.434
8th mode	14.807	14.790	0.115	14.760	0.317
